# Mechanical Recyclability of Polypropylene Composites Produced by Material Extrusion-Based Additive Manufacturing

**DOI:** 10.3390/polym11081318

**Published:** 2019-08-07

**Authors:** Martin Spoerk, Florian Arbeiter, Ivan Raguž, Clemens Holzer, Joamin Gonzalez-Gutierrez

**Affiliations:** 1 Polymer Processing, Montanuniversitaet Leoben, Otto Gloeckel-Straße 2, 8700 Leoben, Austria; 2 Materials Science and Testing of Polymers, Montanuniversitaet Leoben, Otto Gloeckel-Straße 2, 8700 Leoben, Austria

**Keywords:** additive manufacturing, material extrusion, fused filament fabrication, recycling, polypropylene, multiple extrusion, degradation

## Abstract

Due to a lack of long-term experience with burgeoning material extrusion-based additive manufacturing technology, also known as fused filament fabrication (FFF), considerable amounts of expensive material will continue to be wasted until a defect-free 3D-printed component can be finalized. In order to lead this advanced manufacturing technique toward cleaner production and to save costs, this study addresses the ability to remanufacture a wide range of commercially available filaments. Most of them either tend to degrade by chain scission or crosslinking. Only polypropylene (PP)-based filaments appear to be particularly thermally stable and therefore suitable for multiple remanufacturing sequences. As the extrusion step exerts the largest influence on the material in terms of temperature and shear load, this study focused on the morphological, rheological, thermal, processing, tensile, and impact properties of a promising PP composite in the course of multiple consecutive extrusions as well as the impact of additional heat stabilizers. Even after 15 consecutive filament extrusions, the stabilized additively manufactured PP composite revealed an unaltered morphology and therefore the same tensile and impact strength as the initial material. As the viscosity of the material of the 15th extrusion was nearly identical to that of the 1st extrusion sequence, the processability both in terms of extrusion and FFF was outstanding, despite the tremendous amount of shear and thermal stress that was undergone. The present work provides key insights into one possible step toward more sustainable production through FFF.

## 1. Introduction

Today’s society strives for more personalized products that are lightweight and have adequate mechanical properties and a fast but flexible production on demand. Additive manufacturing (AM), also known as three-dimensional (3D)-printing, is one of the few techniques that has the potential to satisfy all of these demands in one manufacturing step [1]. Therefore, AM has prominently gained in importance and has been constantly on the rise year after year [2,3]. Material extrusion, also known as fused deposition modeling (FDM^TM^) or fused filament fabrication (FFF), is an extrusion-based AM technique that has become established in the polymer processing industry as a reliable and inexpensive technique to fabricate prototypes or low quantities of highly complex and/or customized components [2,4]. By selectively depositing thermoplastic filaments in a layer-wise manner according to a predefined computer aided design (CAD) contour, FFF enables the complete flexibility of part design and a prompt fabrication start without the need for extensive process planning or manufacturing of part-dependent molds or tools [5]. However, due to a limited amount of experience with the rather novel AM technologies [6] and still-missing quality standards for the overall process [7], the fabrication of a satisfying, defect-free component in the first processing attempt is almost impossible. 

Apart from deficiencies in mechanical properties that can be the cause for repeatable FFF attempts [8,9,10], mainly, quality insufficiencies and a lack of stability of the FFF process govern such shortcomings. Inadequate choice in FFF parameters [11], e.g., the cooling of the material [12] or an insufficient control of material deposition [13] either due to deficiencies in the filament quality [14] or due to slippage in the printing head [15], can be stated as evident examples. Such issues eventually result in a lack of reproducibility of the part quality over a prolonged production run [2], defects in the internal structure of the 3D-printed part [16], surface imperfections [17], or in the worst case a complete failure of the printing job due to filament buckling or annular backflow [14]. Another common cause for multiple processing attempts for the same component are novel, mostly unknown design constraints, such as the misuse of design parameters such as overhangs, support structures, bridges, cavities [18], or thin walls [19]. An additional underestimated problem is insufficient or over-adhesion during processing, which can result in extensive warpage or even damaged parts [20,21]. In particular, special composites or blend materials can easily suffer from processing instabilities, such as clogging [22] and complex flow and temperature conditions [23], often due to high viscosities [24], nondried filaments [25], or overheated materials [26], which can lead to dimensional inaccuracies and other defects of the parts produced by FFF [27,28,29,30,31,32,33]. Consequently, plenty of expensive material is undesirably wasted until the final component can be successfully processed. 

A practical solution for this problem is to recycle and remanufacture the 3D-printed waste material by shredding the unsuccessfully printed parts, re-extruding them to filaments, and reusing the filaments for further FFF cycles. Apart from the prevalent sustainable benefits of FFF compared to conventional techniques [34], this strategy could lead to additional filament cost reductions, energy savings, and reduced carbon dioxide emissions [35]. Studies on the proposed strategy, however, have been scarce. Tian et al. [36], for example, remanufactured additively manufactured continuous carbon fiber (CF)-reinforced poly(lactic acid) (PLA) once. The authors did not detect a deteriorating effect on the tensile, flexural, or impact properties of 3D-printed specimens, thus highlighting the potential of the proposed remanufacturing strategy. When considering more remanufacturing cycles, though, Cruz Sanchez et al. [37] showed that neat PLA is very prone to material degradation, as the tensile strength of 3D-printed samples deteriorated by approximately 40% after only five remanufacturing cycles. Although plenty of studies have recently investigated the processability of complex recycled regional postconsumer waste by FFF and have even recommended the use of so-called RecycleBots [38,39,40], such as blends of polyethylene [39,41,42,43,44,45], polypropylene (PP) [44,46,47,48,49,50], polyethylene terephthalate (PET) [49,50,51], acrylonitrile butadiene styrene (ABS) [49,52], poly(lactic acid) (PLA) [49,53], polystyrene (PS) [50], polyvinyl alcohol (PVA) [54], or polyamide (PA) [55], the effect of multiple AM and filament extrusion sequences on the processability of materials more complex than the standard FFF material PLA, which is known to be susceptible to thermal degradation [9], has not yet been studied. Moreover, neither the positive effect of thermal stabilizers on retaining mechanical strength [56] nor the degradation of a filler–matrix interface with increasing re-extrusion cycles [57] has been confirmed for advanced composites used in FFF. 

In general, it is understood that both extrusion as well as AM steps play a role in deteriorating the properties of materials susceptible to degradation, such as PLA [37]. Certain processing parameters, such as elevated platform temperatures, long exposure times to high temperatures, or humid atmospheres, undoubtedly influence and therefore complicate the degradation behavior during multiple remanufacturing cycles. However, the establishment of a comprehensive understanding of the underlying degradation processes first requires systematic studies addressing exclusively the filament extrusion step. This approach reduces the number of influencing parameters, while focusing, on the main influencing factor in terms of material degradation due to considerably higher pressures, a higher shear load, and longer residence times during filament extrusion [9].

The present work aims at shedding light onto the complex degradation behavior of advanced thermoplastic composites containing PP, a compatibilizer, and coated mineral fillers by systematically focusing on the effect of multiple filament extrusions on the processability of FFF. As the composite under investigation revealed a more promising thermal stability over time compared to commercially available filaments, the consequences of additional thermal stabilization were studied in terms of processability as well as tensile, impact, morphological, thermal, and rheological properties. Due to the processing stability of the proposed composite, a considerably cleaner production pattern that opens up the pathway for manifold future 3D-printing applications can be recommended. 

## 2. Materials and Methods 

### 2.1. Materials

A polypropylene heterophasic copolymer (PP, Borealis AG, Vienna, Austria) with a tensile strength of 18.6 ± 0.9 MPa [28] was used as the base polymer. The coated mineral filler (MF) FILAFORCE 96A was supplied by Quarzwerke GmbH, Frechen, Germany. To obtain a homogeneous filler distribution within the polymer and a good matrix–filler interface and to prevent thermal degradation, the compatibilizer SCONA TPP 9212 GA (BYK-Chemie GmbH, Wesel, Germany), which is based on PP functionalized with maleic anhydride, and the heat stabilizer Add-Vance TH 130 (BYK-Chemie GmbH, Germany) were used. For the material selection, the aforementioned materials were compared to the following commercially available filaments: black PLA (Prirevo e.U., Grossendorf, Austria), white ABS (Herz Austria GmbH, Schoenberg Austria), HDglass clear polyethylene terephthalate modified with glycol (PETG, Formfutura BV, Nijmegen, the Netherlands), clear poly(methyl methacrylate) (PMMA, Herz Austria GmbH, Austria), and an Arnitel^®^ ID 2045 black thermoplastic copolyester elastomer (TPC, Nexeo Solutions 3D–EMEA, Barcelona, Spain). Unless stated otherwise, all of the mentioned filaments were used as received (not predried). 

### 2.2. Composite Preparation

The composite PP/MF/unstabilized (unstab., Table 1) was mixed in the corotating twin screw extruder ZSK 18 (Coperion GmbH, Stuttgart, Germany) using the following settings: screw speed = 400 rpm, heating zones of the extruder barrel between 200 and 220 °C, length–diameter ratio of the screw = 40. The PP and the compatibilizer were premixed and added at the main hopper, whereas the MF was added by a side-feeder. The extruder was equipped with two atmospheric degassing zones at two locations in the middle of the barrel and one vacuum degassing zone. The extrudate was cooled down in a water bath, pelletized in a strand pelletizer SGS 50-EL (Maag Automatik GmbH, Grossostheim, Germany), and stored under standardized conditions (23 °C air temperature, 50% relative humidity). 

### 2.3. Preparation of Filaments

Both neat PP and PP/MF/unstab., as well as PP/MF/unstab. along with 2 wt % of the stabilizer (designated by PP/MF/stabilized (stab.)), were processed to filaments in the single-screw extruder FT-E20T-MP-IS (Dr. Collin GmbH, Maitenbeth, Germany) with a screw speed of 70 rpm; a die of 1.9 mm in diameter and 25.05 mm in length; the extruder barrel set to 180 °C, 185 °C, and 190 °C; and a nozzle temperature of 200 °C. The extrudate was hauled off through a 3-m-long water bath and through the diameter measurement device Sikora Laser 2010T and an Ecocontrol 600 processor (Sikora AG, Bremen, Germany) and was subsequently spooled to filaments. The Sikora measuring device also calculated the ovality of the filaments. Ovality is defined as the difference between the measured diameter in the vertical direction and the measured diameter in the horizontal direction: therefore, a filament with a perfectly circular cross-section would have an ovality of zero. Prior to the FFF processing or other characterization steps, the filaments were dried for 24 hours at 75 °C und subsequently stored under standardized conditions for at least 72 hours. 

In order to investigate the effect of multiple extrusions, parts of the filaments were pelletized and subsequently re-extruded as described above. This step was repeated 14 times so that a total effect of 15 extrusions could be characterized. Due to the limited material quantity, only for extrusions 1, 2, 3, 5, 7, 10, and 15 was enough filament produced to perform 3D-printing trials. Nevertheless, for all 15 extrusions, tensile tests could be performed on small quantities of filaments. 

### 2.4. Tensile Tests

Tensile tests were performed directly on the filaments under standardized conditions on a Zwick Z001 (Zwick GmbH & Co. KG, Ulm, Germany) with the following settings: initial load = 0.1 MPa, load cell = 1 kN, gauge length = 50 mm, and testing speed = 10 mm·min^−1^. For each re-extrusion and material, seven independent measurements were performed and evaluated to a significance level of 5%. 

### 2.5. Oxidative Induction Time by Differential Scanning Calorimetry

To analyze the activity of the stabilizers, the oxidative induction time was determined by differential scanning calorimetry on a DSC 1 equipped with the gas controller GC 200 (both Mettler Toledo GmbH, Greifensee, Switzerland) based on the standard ASTM D3895. All materials were heated from 25 °C to 200 °C at a heating rate of 20 K·min^−1^ under a nitrogen flow of 50 mL·min^−1^. After a thermal stabilization of 2 min at 200 °C, the gas was switched to oxygen (50 mL·min^−1^). The onset time of oxidation at a constant temperature of 200 °C (oxidative induction time) was evaluated for five filament pieces, each with a mass of 10 ± 1 mg, for each composite. All obtained values were evaluated to a significance level of 5%. 

### 2.6. Preparation of 3D-Printed Specimens

All specimens produced in this study were sliced using the software Simplify3D Version 3.0 (Simplify3D, Cincinati, OH, USA) and produced by means of a Duplicator i3 v2 (Wanhao, Jinhua, China) with a steel nozzle 0.6 mm in diameter using the following FFF settings: nozzle temperature = 220 °C, surrounding temperature = 23 °C, platform temperature = 86 °C, build platform material = PP plate, layer thickness = 0.25 mm, printing speed of the first layer = 14.2 mm·s^−1^, and the printing speed of the remaining layers = 31.2 mm·s^−1^. The flow rate was set in such a way that the printed specimens for the first extrusion revealed a minimal amount of air gaps between adjacent strands/layers, which was pretested by means of optical microscopy (Olympus SZX12, Olympus Life Science Europe GmbH, Hamburg, Germany). For a minimal degree of warpage, the first layer height was set so that maximal adhesion was achieved, while welding of the first deposited layer on the build platform was avoided, as recommended by Reference [20]. Apart from Charpy specimens (Section 2.7), a complex part with small details, overhangs, and a stairstep profile (treefrog geometry taken from the open access platform Thingiverse.com) was used to compare the geometrical stability of the different processing cycles. After the finalization of the printing job, the produced test specimens were detached from the build platform with a spatula and stored under standardized conditions for more than 72 h before subsequent tests were conducted. 

### 2.7. Charpy Tests

The Charpy specimens were printed in a unidirectional orientation (80 × 10 × 4 mm^3^, ISO 179-1 type 1/e/A) in order to determine the effect of the multiple extrusions on the matrix–filler interface. After producing 12 specimens per re-extrusion and material (six specimens per printing job), a notch with a depth of 2 mm and a tip radius of 0.25 mm was introduced by a wedge-shaped blade. The tests were conducted in a randomized order according to the standard ISO 179-1 in an edgewise direction on the impact pendulum Resil 25 (CEAST/Instron, Turin, Italy) at room temperature, and they were evaluated to a significance level of 5%. 

### 2.8. Morphology Analysis

Both the morphology of cryofractured filaments and the fracture surfaces of Charpy specimens prepared by FFF that had been previously sputtered with gold for 180 s at 20 mA were investigated by means of scanning electron microscopy (SEM) on a Tescan Vega II (Tescan Brno s.r.o., Brno, Czech Republic) at 5 kV using secondary electrons. 

### 2.9. Rheology

Viscosity data of the re-extruded filaments were obtained from 3D-printed discs 25 mm in diameter and 2 mm in thickness by using the rotational rheometer Physica MCR 501 (Anton Paar GmbH, Graz, Austria) in a plate–plate measuring geometry. The tests were conducted under a nitrogen atmosphere at the nozzle temperature (*T_N_*) used during FFF (*T_N,PP_* = *T_N,PP/MF/stab_*_._ = *T_N,PP/MF/unstab._* = 220 °C) or at one recommended from the literature or the supplier’s data sheet (*T_N,PLA_* = *T_N,ABS_* = *T_N,PMMA_* = *T_N,TPC_* = 250 °C [9] and *T_N,PETG_* = 230 °C). All measurements were performed at a gap of 1 mm in oscillatory shear-controlled mode in the materials’ linear viscoelastic region (at 0.3% deformation). For the time sweep measurements, the materials were sheared at an angular frequency of 100 rad·s^−1^ for 30 min, because during FFF, shear rates ≥100 rad·s^−1^ arise, as recommended by Reference [9]. For the frequency sweep measurements, at least three independent measurements per re-extrusion and material were measured between angular frequencies of 500 and 0.1 s^−1^. The results are presented as averaged viscosity curves evaluated to a significance level of 5%. 

### 2.10. Thermogravimetric Analysis

Thermogravimetric analysis (TGA) was performed in a TGA/DSC 1 (Mettler-Toledo GmbH, Greifensee, Switzerland) with crucibles made of aluminum oxide with a volume of 30 μL. All samples were exposed to a heat run between 25 and 600 °C at a heating rate of 10 K·min^−1^. The final temperature of 600 °C was held for 10 min. Measurements were performed on the filaments, which each had an initial mass of 12.5 ± 2.5 mg, under a constant oxygen flow of 5 mL·min^−1^, since the re-extrusion process was done under atmospheric conditions. Five repetitions were performed on each material, and the results were evaluated to a significance level of 5%. 

**Figure 1:**
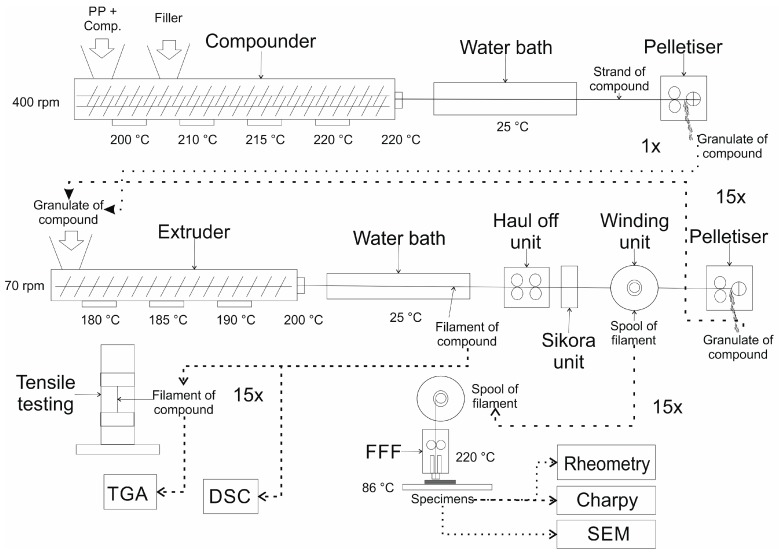
Schematic representation of the complete experimental procedure.

A complete overview of the experimental procedure is schematically shown in Figure 1.

## 3. Results and Discussion

### 3.1. Tendency of Thermal Degradation of Commercially Available Filaments

**Figure 2:**
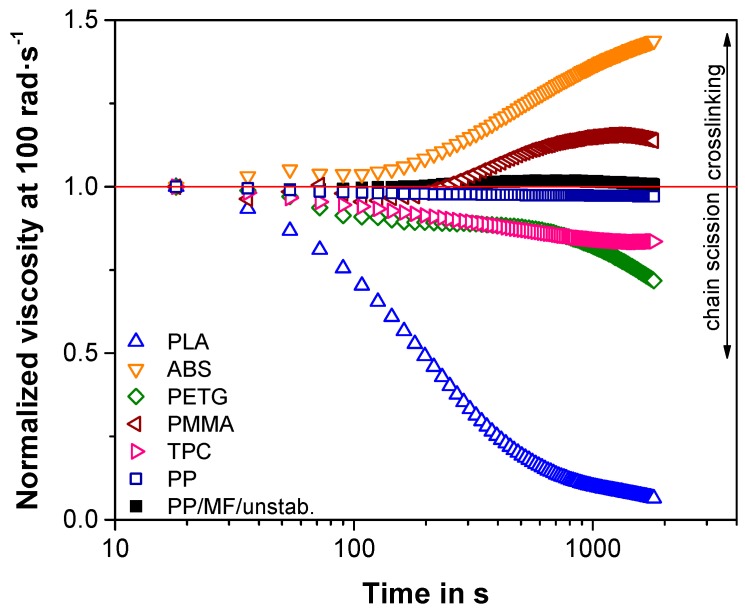
Time sweep rheological measurements for frequently used, commercially available materials for fused filament fabrication (FFF) and for the composite PP/MF/unstab. and its base polymer PP, measured at their suggested nozzle temperature. The red line at a normalized viscosity of 1 represents a visualization of ideal thermal stability. As highlighted, above this line, crosslinking occurs, and below this line, chain scission takes place.

It is known from Reference [9] that rheological time sweep measurements are a useful tool in determining the thermal stability over time for 3D-printing applications. To compare the thermal stability under constant shear of different materials used in FFF, such time sweep measurements are represented in a way that normalizes the viscosity by the first viscosity value measured at 18 s for each material (Figure 2). The ester-based commercially available polymers PETG, TPC, and in particular PLA reveal a prompt decrease in viscosity after a few seconds of shearing due to chain scission [58]. The undried state of the polyesters can especially cause a drastic deterioration in viscosity [59]. Although such viscosity decreases are known not to influence the printing quality due to very short residence times in the hot end of a 3D printer [9], multiple extrusion sequences do cause high shear during longer times. Consequently, this finding can be the reason for the deteriorating trend in mechanical properties of PLA for multiple extrusions [37]. However, this also means that both PETG and TPC might reveal slightly decreasing mechanical properties for multiple filament extrusion sequences due to the risk of chain scission and a decrease in the degree of crystallinity [60]. 

Interestingly, both PMMA and ABS exhibited an increasing viscosity for longer shearing times (Figure 2). Especially for ABS, the morphology seemed to change drastically, as the viscosity kept on rising constantly and did not stagnate in a plateau, such as for PMMA. This was most certainly caused by the thermal degradation of the elastomeric polybutadiene phase of ABS, which tends to degrade by producing hydroperoxide radicals, which in turn leads to facilitated crosslinking reactions [61], especially at temperatures above 220 °C [62]. Pure PMMA thermally degrades by chain scission starting at temperatures of roughly 200 °C. However, the thermal stabilizers that are used to prevent full depolymerization of the main polymeric chain can promote crosslinking to a certain extent [63]. This might explain the small viscosity increase followed by a plateau in the time sweep (Figure 2). Although crosslinking reactions induced after processing show the potential to improve the toughness and strength as well as decrease the anisotropy of parts produced by FFF [64], crosslinking reactions during processing, e.g., during re-extrusions of 3D-printed components, can deteriorate the processability by means of extrusion and FFF, as long as the materials are not reversibly crosslinked [65]. Hence, re-extrusions of commercially available ABS and PMMA might only be realizable for a limited amount of re-extrusion sequences. Multiple filament extrusions can additionally be problematic for ABS, as during printing the amount of rubbery butadiene phase can already be decreased, and therefore its mechanical properties can drastically change [66]. 

As already pointed out by Jagenteufel et al. [62], PP was considerably more stable under constant shear and temperature than the previously discussed polymers (Figure 2). The same was valid for the composite PP/MF/unstab., which hinted at revealing a homogeneous filler distribution over time and a thermally stable matrix–filler interface. As the PP-based composite did not appear to degrade through chain scission or crosslinking (Figure 2), the material should serve well for investigating the effect of multiple extrusions on the behavior of 3D-printed parts. Additionally, the incorporation of the mineral filler facilitated the processability of the PP composite compared to neat PP due to a drastic improvement in the dimensional accuracy, particularly the shrinkage and warpage of the 3D-printed parts [28,29,30,31]. Therefore, the following sections only address the properties of additively manufactured parts of the PP-based composites PP/MF/unstab. and PP/MF/stab.

### 3.2. Tensile Test Results

**Figure 3:**
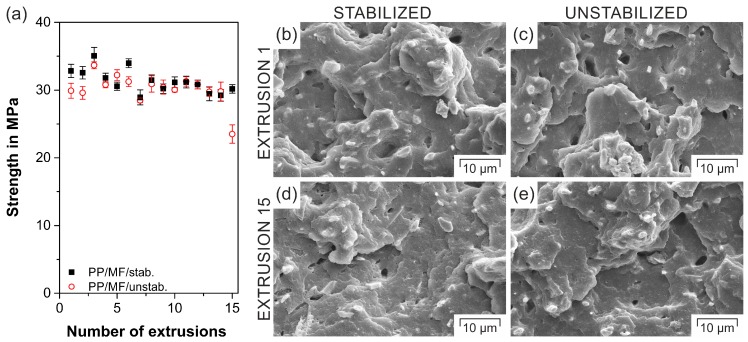
Filament tensile strength as a function of the number of extrusions for both composites (**a**); and scanning electron microscopy images of the cryofractured filaments of PP/MF/stab./E-1 (**b**), PP/MF/unstab./E-1 (**c**), PP/MF/stab./E-15 (**d**), and PP/MF/unstab./E-15 (**e**).

Independent of the number of extrusions, both composites (Figure 3) exhibited an approximately 50% higher strength than neat PP (20.9 ± 0.8 MPa [31]) due to the addition of high-aspect ratio fillers. Besides some minor strength fluctuations, which were also observed for multiple extrusions of PP filled with montmorillonites [67], the strength of the composite PP/MF/stab. appeared to be roughly independent of the number of extrusion sequences, similarly to comparable studies on neat PP [68] and PP composites [67]. This trend hinted at a sufficient stabilization of the polymer by the additional heat stabilizers, which could also be confirmed by an oxidative induction time higher than 60 min independent of the number of extrusions (Table 2). For the unstabilized composite PP/MF/unstab., a similar trend could be found. However, a drastic drop in strength of more than 20% could be discerned for this material after extrusion 14. This decrease was connected to a reduction in the activity of the stabilizer that was added from the material supplier, as can be seen from the significant drop in the oxidative induction time of the unstabilized composites (Table 2). As a consequence, the molecular weight of PP decreased, leading to a reduction in its viscosity (Section 3.5) as well as its tensile strength [69]. The fact that a rather mild shear screw setup was chosen for the filament extrusion [70] and that the degradation is strongly dependent on the type of polymerization technology [71] may explain the unexpectedly late strength deterioration. The slight strength variations in one material, particularly within the first six extrusions, may be explained by other influencing factors such as the degree of crystallinity, which usually increases with more re-extrusion steps [68,72]. The minimal differences in strength between the two composites within the first three extrusions could have been caused by the slightly different overall amount of filler due to the addition of 2 wt % of stabilizer to the composite PP/MF/unstab. during processing. 

The morphology of the 1st (Figure 3b,c) and the 15th extrusions (Figure 3d,e) of both composites did not significantly change. All investigated composites revealed a homogeneous filler distribution and a strong matrix–filler interface. Apart from the good contact between the filler and the matrix, this was additionally proven by large torn-out segments in the cryofracture that were caused by fillers encapsulated by the matrix, similarly to Reference [29]. In contrast to References [57,67], this independency of the morphology on the number of extrusions proved that the filler–matrix interface did not tend to deteriorate, which additionally explained the retention of the original strength even after many sequences of re-extrusions. Moreover, such high strength values hinted at a decent processability by means of FFF [30], even for multiple re-extrusions. 

### 3.3. Impact Results

**Figure 4:**
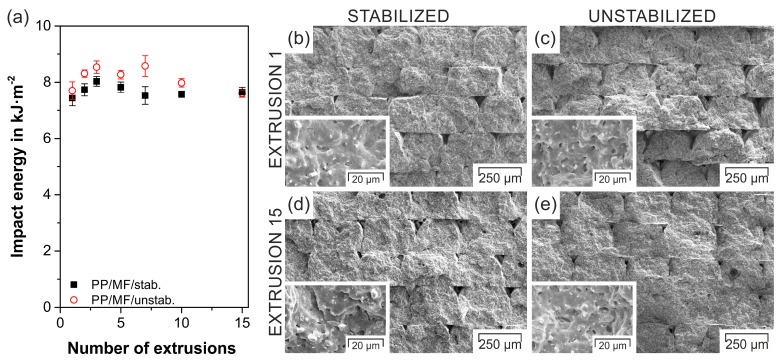
Impact energy of notched Charpy specimens produced by FFF as a function of the number of extrusions for both composites (**a**); and scanning electron microscopy images of the impact fracture surfaces of PP/MF/stab./E-1 (**b**), PP/MF/unstab./E-1 (**c**), PP/MF/stab./E-15 (**d**), and PP/MF/unstab./E-15 (**e**).

Compared to neat PP (30.4 ± 2.8 kJ·m^−2^, [28]), the investigated composites revealed an approximate reduction of 75% in impact energy (Figure 4a) despite the filler’s acicular shape, as the fillers acted as initiation points for defects and additionally decreased the impact fracture area [73]. Due to the extrusion and FFF process, the mineral fillers were oriented in a flow/printing direction [31,74] and were consequently perpendicular to the impact direction, which can be seen in the inserts of the respective fracture surface (Figure 4b–d). Therefore, the slightly higher filler content of PP/MF/unstab. induced by the processing sequence resulted in a more effective crack deflection and hence an elevated absorbed impact energy [73]. Similarly to neat PP [67,68,72], the impact energy was not highly dependent on the number of extrusions (Figure 4a). For both composites, no decrease in the impact energy at more re-extrusion steps, as was observed for PP filled with montmorillonites [67], could be discerned, as the filler–matrix interface did not visibly degrade for higher numbers of extrusions (inserts in Figure 4d,e). In fact, a slight trend of improved impact energy at more extrusion steps (up to three extrusions) was observed. This finding was in accordance with similar multiple extrusion studies on polypropylene clay composites [72] and can be explained by a marginal, but not visible, increase of the filler dispersion due to the longer residence time in the extruder at more extrusion steps [72]. In contrast to 3D-printed continuous CF-reinforced PLA composites [36], for which the fracture mechanism changed from fiber pullout to fiber breakage after one remanufacturing step, which also led to an increase in the impact strength, in the present case, neither the morphology nor the fracture surface underwent a drastic change when being extruded multiple times (compare Figure 4b to Figure 4d and Figure 4c to Figure 4e). The amount of air gaps in between the deposited strands, which can drastically influence the impact energy of parts produced by FFF [30], was low and comparable among the investigated fracture surfaces. Only the fracture surface of the unstabilized composite exhibited a weak reduction in inter- and intralayer voids at increasing extrusion steps (Figure 4e), similarly to remanufactured neat PLA processed by FFF [37]. As addressed by Cruz Sanchez et al. [37], the observed larger cross-flow could be caused by a decreased melt viscosity due to the lack of stabilizer, which will be elaborated on in detail in Section 3.5. 

### 3.4. Processability

**Figure 5:**
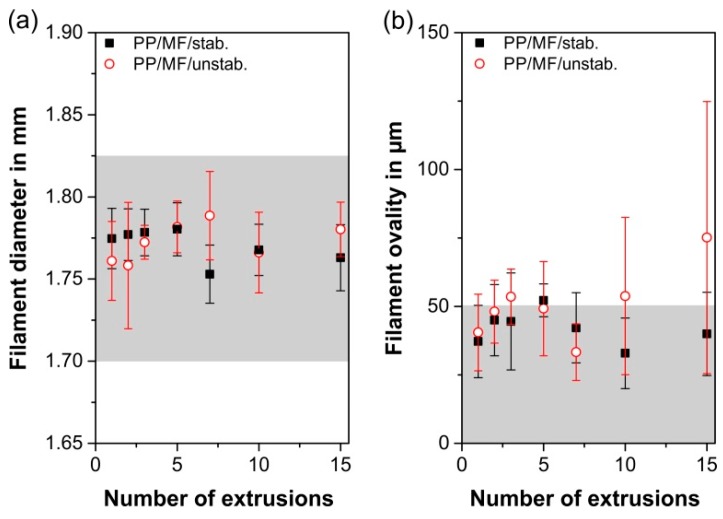
Filament diameter (**a**) and ovality (**b**) as a function of the number of extrusions for both composites. The gray area determines the threshold for a 3D print with decent quality for the 3D-printer used.

As expected from the sufficient strength of all filaments (Section 3.2) and the monitored filament diameters (Figure 5a), which were independent from the numbers of extrusions within the diameter tolerances of the 3D printer used (1.700–1.825 mm, gray area in Figure 5a), almost all investigated filaments were flawlessly printable. As particularly the flow rate during material extrusion-based AM is affected by the ovality of the filaments, for ideal processability, the filament ovality should stay below 50 µm [30], which was nearly realizable on the composite PP/MF/stab. for all extrusions (Figure 5b). However, for PP/MF/unstab., the filament ovality tended to increase at higher extrusion sequences. Particularly the ovality of the 15th extrusion was too high and fluctuated too much for a decently appearing 3D-printed part due to the associated flow rate variabilities. The rheological differences that caused this increase in ovality will be discussed in detail in Section 3.5. 

**Figure 6:**
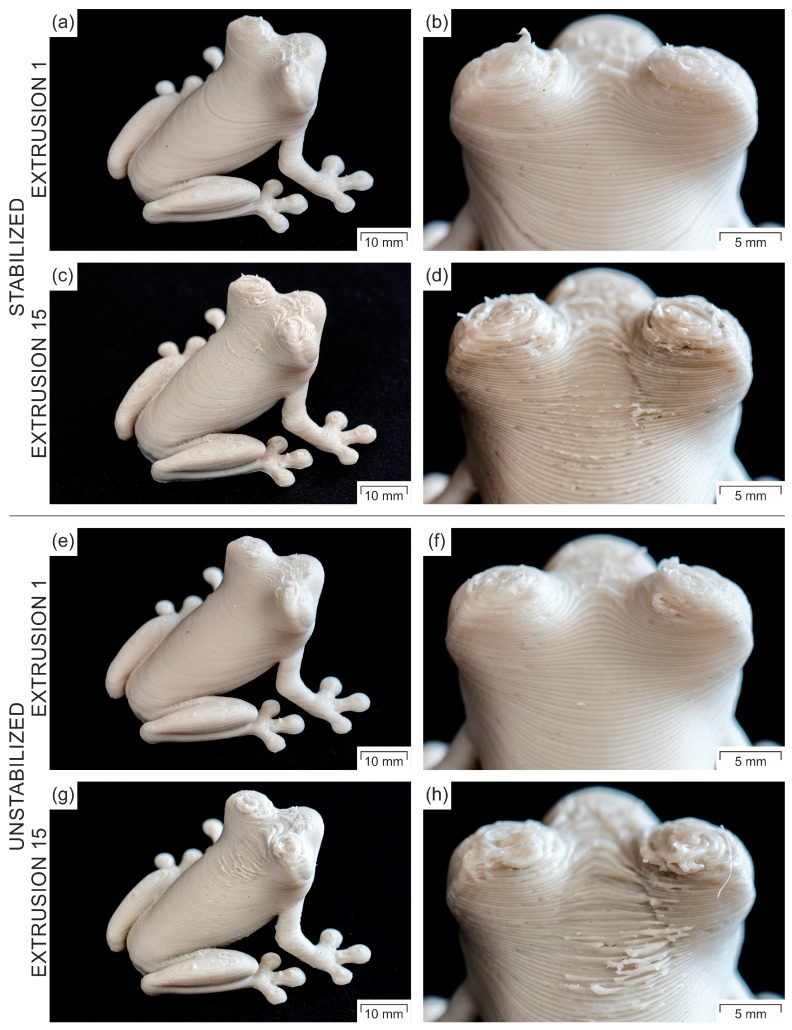
Comparison of the 3D-printing quality of a complex part with distinct overhangs, small details, and stairstep profiles for the two composites extruded 1 and 15 times.

As the additively manufactured Charpy specimens were not complex enough to validate the consequences of the high filament ovality, a complex part with small details, overhangs, and a stairstep profile was processed, and the printed parts of both materials with different extrusion sequences were compared (Figure 6). Due to the sufficient quality of the filament diameter and ovality, the parts of both composites resulting from the first extrusion (Figure 6a,e) exhibited a comparably good printing quality without any problems in connection to oozing, overhangs, warpage, or the display of small details (Figure 6b,f). The part resulting from the 15th extrusion of PP/MF/stab. (Figure 6c,d) revealed only slight visual deficiencies in terms of minor flow rate fluctuations. Nevertheless, apart from the expected minor change in color [75], the printing quality of this composite was still more than decent, despite the tremendous shearing to the material introduced during filament re-extrusion. On the contrary, the part resulting from the 15th extrusion of PP/MF/unstab. (Figure 6g,h) showed considerably worse printing quality than all the other investigated printed parts did, particularly in terms of surface quality, oozing, and representing small details. All of these visual deficiencies were mainly based on flow rate instabilities due to the aforementioned high filament ovalities and ovality deviations (Figure 5b). 

### 3.5. Rheology Results

**Figure 7:**
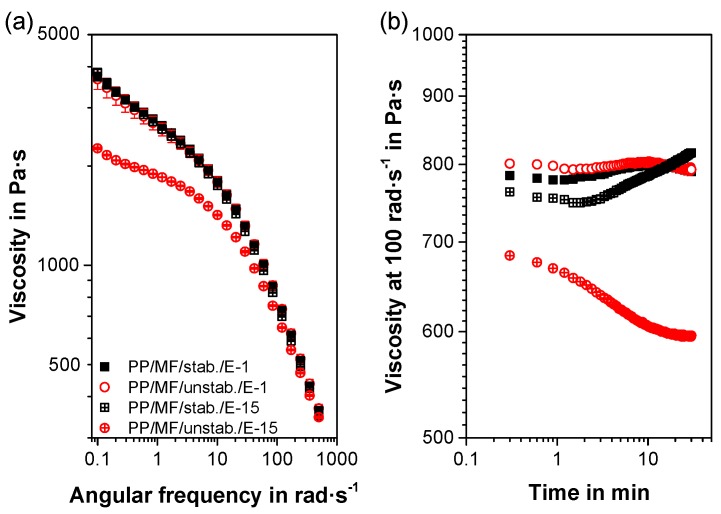
Mean frequency sweep and error bars of three individual measurements (**a**) and time sweep (**b**) for the two composites extruded 1 and 15 times, measured at 220 °C.

As expected from the similar composition and the minimal time/amount of shearing, the shear viscosity curves of the first extrusions of both composites were nearly identical (Figure 7a). The 15th extrusion of PP/MF/stab. exhibited an indifferent shear viscosity curve, too. However, the 15th extrusion of PP/MF/unstab. revealed a considerably reduced viscosity compared to the other materials, in particular at lower shear rates. This significant drop in viscosity was caused by the extensive thermal degradation of 15 extrusion steps and the lack of additional stabilizers [76], which was also visible in the reduced oxidative induction time (Table 2). Without additional thermal stabilization, the macromolecules started to degrade by chain scission with increasing numbers of extrusions [77]. As a result, the molecular weight decreased [57,71,77,78,79], the melt flow rate rose [71,76,78,80], the shear viscosity at low shear rates was reduced [57,67,72], and the elongational viscosity or melt strength decreased [81,82,83]. The decreased viscosity (shear and elongational) was the main reason for the ovality deflections of higher extrusion numbers of PP/MF/unstab. When the filament of the unstabilized composite was extruded, the extrudate sagged more due to its lower melt strength, which led to an oval geometry before the extrudate came in contact with the water bath during filament extrusion [84]. 

The overall differences in the viscosities in the time sweeps (Figure 7b) were marginal due to the low viscosity differences at higher shear rates. As expected from the similarities in the viscosity curves, the time sweeps of both the 1st extrusion and the 15th extrusion of PP/MF/stab. were in a similar viscosity range and stayed nearly constant over time. Only the viscosity of the 15th extrusion of PP/MF/stab. seemed to slightly increase over time, which could have been due to the formation of shear-induced filler agglomerations. A similar effect could be seen in the rather steep increase in the viscosity of all materials for smaller shear rates in Figure 7a due to the measurements of shear rates starting from high to low, similarly to Reference [72]. In contrast, the 15th extrusion of PP/MF/unstab. revealed both an initial viscosity that was approximately 100 Pa·s lower than the other compounds and a decreasing viscosity over time. This finding additionally confirmed the susceptibility of the unstabilized composite to thermal degradation by chain scission, as described above. 

### 3.6. Thermal Gravimetric Analyses

**Figure 8:**
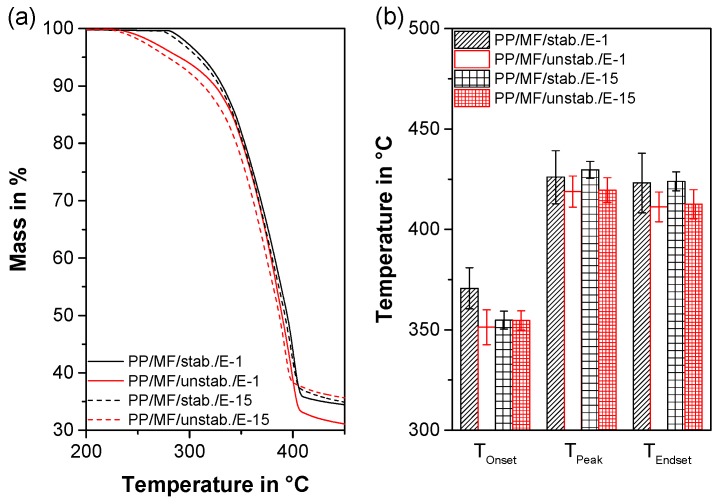
Mean mass as a function of temperature (**a**) and a detailed comparison of the onset (*T_Onset_*), peak (*T_Peak_*), and end-set temperature (*T_Endset_*) for both composites, each extruded once and 15 times (**b**).

To investigate the impact of thermo-oxidative degradation, the 1st and 15th extrusion of both composites were investigated by means of TGA. At first glance, the averaged thermograms of the different materials appeared very similar (Figure 8a). However, upon a closer look (Figure 8b), it could be seen that for the first extrusion, the composite PP/MF/unstab. started to decompose at significantly lower temperatures (*T_Onset_* = 351.3 ± 8.7 °C) than PP/MF/stab. (*T_Onset_* = 370.7 ± 10.2 °C) did. The *T_Peak_* and *T_Endset_*, though, did not show any differences between the first extrusions of the materials. This finding showed that after one extrusion step there was already a slight difference between the stabilized and unstabilized material in terms of the initiation of thermo-oxidative degradation, similarly to the oxidative induction times (Table 2), while the final decomposition steps for the first extrusion were not influenced by the stabilizer. Interestingly, the *T_Onset_* of the 15th extrusion of PP/MF/stab. was also reduced, similarly to what was described above, whereas the *T_Onset_* of the 15th extrusion of PP/MF/unstab. was maintained at the same level to that of the 1st extrusion. The *T_Peak_* and *T_Endset_*, however, tended to decrease slightly after 15 extrusions of PP/MF/unstab. compared to PP/MF/stab. This finding revealed that the unstabilized compounds started decomposing at similar or marginally lower temperatures, but decomposed at a slightly faster rate. All in all, the overall differences in terms of oxidative degradation were very little. Consequently, it can be concluded that none of the composites, not even after 15 extrusions, was significantly susceptible to thermo-oxidative degradation. 

## 4. Conclusions

In summary, the present study demonstrates the susceptibility to thermal degradation of commercially available filaments for material extrusion-based additive manufacturing. As a promising alternative, an advanced polypropylene composite containing 15 vol % of coated mineral fillers and a compatibilizer is proposed. For standard printing parameters, advanced materials, and small 3D-printed parts, the filament production step by extrusion is seen as the major influencing factor on material degradation behavior due to the harsh processing conditions (elevated shear rates, pressures, and residence times). Thus, by analyzing the consequences of multiple filament extrusions on tensile, impact, rheological, morphological, and thermal properties as well as on the processability of this novel composite, the applicability of a sustainable processing strategy, which may finally include the recycling and remanufacturing of 3D-printed waste material, was assessed. Despite (up to) 15 extrusion steps, the composite without additional heat stabilizers still revealed a strong filler–matrix interface, a homogeneous filler distribution, and a stable filament diameter. However, due to the lack of additional stabilizers and a reduction in the initial stabilizers, the polymer started to slightly degrade by chain scission after 15 extrusions. As a consequence, the filament strength decreased by ~20%, and the low shear viscosity was significantly reduced by ~40%. In turn, the filament exhibited a considerably higher ovality, which led to flow rate instabilities during FFF and therefore a deteriorated surface and overall printing quality for complex components. By adding heat stabilizers in the first extrusion step, the polymer’s tendency to thermally degrade was nearly diminished. Consequently, even after 15 extrusion sequences, the morphology, viscosity, tensile strength, impact energy, and process quality, both by extrusion and FFF, remained unaltered compared to the nondegraded first extrusion. 

Our findings offer valuable insights into the applicability of the proposed remanufacturing strategy for 3D-printed waste by focusing on the impact of the extrusion step as the key factor in material degradation. For certain suggested filament types, the realization of this strategy can enable a sustainable production pattern that offers significant material and cost savings for a broad audience. Future work should address the influence of varying printing parameters, e.g., platform temperatures, exposure times to high temperatures, and different atmospheres, on the recyclability of additively manufactured materials. 

## Figures and Tables

**Table 1:** Composition and designation of the compound, consisting of polypropylene (PP), the compatibilizer (Comp.), and the filler.

Sample Designation	PP (vol %)	Comp. (vol %)	Filler (vol %)
PP/MF/unstab.	83.7	1.3	15.0

**Table 2:** Oxidative induction time at 200 °C for the composites investigated.

Sample Designation	Oxidative Induction Time (s)
PP/MF/stab./E-1	>3600
PP/MF/stab./E-15	>3600
PP/MF/unstab./E-1	213.0 ± 22.7
PP/MF/unstab./E-15	131.2 ± 18.3
